# Neurotrophin receptors expression in mesial temporal lobe epilepsy with and without psychiatric comorbidities and their relation with seizure type and surgical outcome

**DOI:** 10.1186/s40478-014-0081-2

**Published:** 2014-07-16

**Authors:** Ludmyla Kandratavicius, Jaime Eduardo Hallak, Carlos Gilberto Carlotti, Joao Alberto Assirati, Joao Pereira Leite

**Affiliations:** Ribeirao Preto School of Medicine, Department of Neurosciences and Behavior, University of Sao Paulo (USP), Av Bandeirantes 3900, CEP 14049-900 Ribeirao Preto, Brazil; Center for Interdisciplinary Research on Applied Neurosciences (NAPNA), USP, Ribeirao Preto, Brazil; National Institute of Science and Technology in Translational Medicine (INCT-TM – CNPq), Ribeirao Preto, Brazil; Ribeirao Preto School of Medicine, Department of Surgery, USP, Ribeirao Preto, Brazil

**Keywords:** Temporal lobe epilepsy, Psychosis, Major depression, Neurotrophin receptor, TrkB, p75NTR, Psychiatric comorbidity

## Abstract

Epilepsy and psychiatric comorbidities are frequently associated, but their common biological substrate is unknown. We have previously reported altered structural elements and neurotrophins (NTs) expression in mesial temporal lobe epilepsy (MTLE) patients with psychiatric comorbidities. NTs receptors can regulate neurotransmission and promote neuroplasticity, being important candidates in the regulation and manifestation of psychopatological states and seizure-related events. MTLE hippocampi of subjects without psychiatric history, MTLE + major depression, MTLE + interictal psychosis derived from epilepsy surgery, and control necropsies were investigated for p75^NTR^, TrkB, TrkA, and TrkC immunohistochemistry. Increased expression of p75^NTR^, decreased TrkA, unaltered TrkC, and complex alterations involving TrkB expression were seen in MTLE groups. Increased TrkB expression in patients without complete seizure remission and in those with secondarily generalized seizures was seen. Decreased p75^NTR^ expression associated with interictal psychosis, and increased TrkB in those with psychosis or major depression was also reported, although their p75^NTR^/TrkB ratios were lower than in MTLE without psychiatric comorbidities. Our results provide evidence of alterations in expression of NTs receptors in the epileptogenic hippocampus that are differentially modulated in presence of psychiatric comorbidities. As already explored in animal models, even in chronic human MTLE increased TrkB expression, among other NT receptors alterations, may play a major role in seizure type, frequency and surgery outcome.

## Introduction

Psychiatric comorbidities are frequent in epileptic patients, although the precise nature of this association is still matter of debate [1,2]. Mesial temporal lobe epilepsy (MTLE), the most common cause of intractable epilepsy in adults, usually shows hippocampal sclerosis (HS), neuronal loss, gliosis and mossy fiber sprouting [3–6]. We have recently shown neuropathological data suggesting that there is a structural basis for psychiatric symptoms in the hippocampus of MTLE patients [7,8], and altered neurotrophin (NT) expression in presence of psychiatric comorbidities [9].

Hippocampal NTs are upregulated in MTLE patients [9,10] and in animal models of epilepsy [11–16]. NT receptors have been shown to play important roles in epileptogenesis, especially tyrosine receptor kinase B (TrkB) and p75 neurotrophic receptor (p75^NTR^) [17–19]. In human MTLE, a qualitative study performed in the dentate gyrus and Ammon’s horn showed increased p75^NTR^ expression in glia and remaining neurons, and few alterations in Trk expression [20]. In animal models of epilepsy, increased NT receptors expression has also been related to increased excitability [21–25].

In schizophrenia, decreased TrkB and TrkC expression was reported in the prefrontal cortex [26–28], although truncated forms of TrkB were elevated [29]. In hippocampus, decreased TrkA and TrkB expression has been documented [27,30], as well as no change in TrkB [31]. Immunolabeling and mRNA levels of TrkA and TrkC were decreased in the hippocampus of suicide subjects with major depression when compared to normal subjects and p75^NTR^ was increased [32]. In post-mortem brain of teenage suicide victims TrkB mRNA levels were decreased in both prefrontal cortex and hippocampus [33].

Structural abnormalities, altered neuronal plasticity and excitability have been described in epilepsy and psychiatric disorders [for review please see [2,34]], and such features are intrinsically regulated by NTs and their receptors [35,36]. Here we described NT receptors expression in subfields of the hippocampus, subicular complex and entorhinal cortex in a series of MTLE patients with and without comorbid major depression and interictal psychosis. Finally, we investigated the possible correlation between NT receptors expression, seizure type and outcome, and further clinical characteristics.

## Materials and methods

### Patients

We analyzed the hippocampal formation from MTLE specimens freshly collected in the operating room and non-epileptic controls from necropsy, collected between 3.5-6 hours after death. A < 24 h postmortem time limit allows comparison of necropsy tissue with freshly collected surgical specimens for their protein levels, cell morphology and tissue integrity [37,38]. This study followed the principles of the Declaration of Helsinki, was registered in Brazilian’s Health Ministry and was approved by the Research Ethics Committee of our institution, where this study was performed (process HCRP #10180/2007). Written informed consent was obtained from all patients used in this study, and the Research Ethics Committee also approved the Consent Term.

MTLE specimens were derived from 40 MTLE patients who underwent a standard en bloc anterior temporal resection (including 3–4 cm of the hippocampus) for medically intractable seizures. All had clinical neuropathological confirmation of HS. They were divided into 3 groups: 14 MTLE patients without any history of psychiatric disorder (MTLE group); 13 MTLE patients with interictal psychosis (MTLE + P group); and 13 MTLE patients with a diagnosis of major depression (MTLE + D group). A summary of clinical characteristics of all groups is depicted in Table 1. According to Blümcke’s HS categories [39], 17 patients had severe HS, 16 classical HS, 5 CA1 HS and 2 CA4 HS. No differences were seen among MTLE groups regarding HS categories (for details, please see neuropathological data in Table 2). For comparison purposes, ten human non-epileptic control hippocampi from necropsies were processed and analyzed in the same manner as the surgical cases. Underlying diseases causing death were cardiomyopathy, pulmonary infarct or renal-hepatic failure, with no history of hypoxic episodes during agony, seizures or neurological diseases. Furthermore, there was no evidence of brain pathological abnormalities on clinical postmortem examination of the mesial temporal structures. MTLE and control specimens were collected between 1996 and 2006. As discussed elsewhere [8], the number of interictal psychosis cases (n = 13) collected within this timeframe corresponds to almost the totality of interictal psychosis cases operated in our Epilepsy Surgery Center in this same period.

**Table 1:** **Demographic and clinical data**

	**MTLE**	**MTLE + D**	**MTLE + P**	**Controls**	***Statistics***
Male (*n*)	8	5	7	4	*No difference*
Female (*n*)	6	8	6	6	
IPI present (*n*)	9	8	7	n.a.	*No difference*
IPI absent (*n*)	5	5	6	n.a.	
Febrile seizure present in IPI (*n*)	5	3	5	n.a.	*No difference*
Febrile seizure absent in IPI (*n*)	4	3	1	n.a.	
Febrile seizure unknown (*n*)	0	2	1	n.a.	
Age of first seizure (years)	3.0 ± 2.3	4.3 ± 5.5	6.8 ± 8.4	n.a.	*No difference*
Age when seizures became recurrent or age of onset (years)	10.2 ± 6.4	13.0 ± 8.1	13.8 ± 8.4	n.a.	*No difference*
Seizure type: CPS (*n*)	6	1	4	n.a.	*No difference* ^***tr***^
Seizure type: SGS (*n*)	8	12	9	n.a.	
Seizure frequency (monthly)	12.7 ± 10.1	17.2 ± 15.6	11.7 ± 10.1	n.a.	*No difference*
Right HS (*n*)	6	7	5	n.a.	*No difference*
Left HS (*n*)	6	4	6	n.a.	
Bilateral HS (*n*)	2	2	2	n.a.	
Right handedness (*n*)	12	11	13	n.a.	*No difference*
Left handedness (*n*)	1	2	0	n.a.	
Bilateral handedness (*n*)	1	0	0	n.a.	
Memory in verbal tasks: average or above (*n*)	3	7	2	n.a.	*No difference*
Memory in verbal tasks: below average (*n*)	11	6	11	n.a.	
Memory in non-verbal tasks: average or above (*n*)	9	7	3	n.a.	*No difference* ^***tr***^
Memory in non-verbal tasks: below average (*n*)	5	6	10	n.a.	
Full scale IQ	82.2 ± 7.9	84.3 ± 9.6	77.2 ± 7.3	n.a.	*No difference*
Years at school	6.2 ± 3.0	5.1 ± 3.6	4.2 ± 2.6	n.a.	*No difference*
Age at surgery (or at death for controls) (years)	36.1 ± 4.7	37.1 ± 5.4	38.0 ± 6.0	48.1 ± 18.9	*No difference*
Duration of epilepsy (years)	25.9 ± 6.4	24.1 ± 8.9	24.1 ± 8.6	n.a.	*No difference*
Collected side: right (*n*)	7	8	6	6	*No difference*
Collected side: left (*n*)	7	5	7	4	
Collected side: left (*n*)	7	9	6	4	
Surgical outcome: complete remission of seizures (*n*)	8	2	4	n.a.	***Fisher’s Exact Test, p = 0.046: MTLE × MTLE + D***
Surgical outcome: only auras and/or fewer seizures (*n*)	6*	11*	9	n.a.	

Values indicated as mean ± std. deviation when applicable. A statistical trend (***tr***: 0.05 ≤ p ≤ 0.07) was observed suggesting a higher proportion of MTLE + D patients with SGS than MTLE, and a higher proportion of MTLE + P patients with worse performance in non-verbal memory tasks than MTLE.

*: Two patients in MTLE group; one patient in MTLE + D, and four patients in MTLE + P group presented with auras and seizures post-surgery; the remaining had only auras, and the proportion was increased in the MTLE + D group. IPI: initial precipitant injury; CPS: complex partial seizure; SGS: secondarily generalized seizures; HS: hippocampal sclerosis; n.a.: not applicable.

**Table 2:** **Summary of neuropathological data**

	**MTLE**	**MTLE + D**	**MTLE + P**	**Controls**
**Neuronal density (#/mm** ^**3**^ **× 10** ^**3**^ **)**				
Granular layer	87.1 ± 30.5	87.7 ± 36.6	89.9 ± 34.0	171.5 ± 39.5
Hilus	4.8 ± 3.4	5.1 ± 4.3	5.6 ± 3.9	13.9 ± 5.9
CA4	10.6 ± 7.9	8.8 ± 7.5	7.9 ± 4.1	23.6 ± 8.2
CA3	15.1 ± 10.5	11.7 ± 9.4	14.8 ± 8.9	22.9 ± 6.4
CA2	17.7 ± 10.3	15.9 ± 5.4	11.9 ± 5.8	22.8 ± 8.2
CA1	7.9 ± 5.4	3.7 ± 2.5	2.9 ± 2.4	18.3 ± 8.1
Prosubiculum	11.4 ± 6.1	7.8 ± 4.2	6.2 ± 4.0	23.7 ± 6.2
Subiculum	21.5 ± 9.7	20.0 ± 12.6	16.0 ± 10.6	17.6 ± 7.4
Parasubiculum	17.3 ± 8.1	14.9 ± 8.3	15.3 ± 8.3	17.3 ± 5.6
Entorhinal cortex	27.4 ± 11.3	20.0 ± 8.5	20.8 ± 5.8	38.6 ± 8.6
**HS classification (** ***n*** **)**				
Severe HS	7	5	5	n.a.
Classical HS	5	6	5	n.a.
CA1 HS	1	2	2	n.a.
CA4 HS	1	0	1	n.a.
**p75** ^**NTR**^ **IR area (μm** ^**2**^ **× 10** ^**2**^ **)**				
Granular layer	3974.2 ± 1425.8	2945.2 ± 1254.1	3064.6 ± 1135.7	1586.7 ± 623.4
Hilus	1961.7 ± 585.5	1708.5 ± 532.3	1721.9 ± 558.6	1115.7 ± 359.1
CA4	2570.1 ± 743.2	1853.3 ± 702.5	2350.2 ± 619.5	1224.4 ± 418.1
CA3	2950.6 ± 693.7	3816.7 ± 1342.0	2756.6 ± 791.6	1964.5 ± 786.6
CA2	4189.7 ± 924.4	3817.4 ± 1085.0	3598.5 ± 726.5	2242.7 ± 910.7
CA1	2941.2 ± 916.4	2660.8 ± 663.0	2583.7 ± 260.7	1572.0 ± 692.3
Prosubiculum	2747.7 ± 896.4	2243.4 ± 568.8	2278.7 ± 529.2	1652.9 ± 810.6
Subiculum	3372.1 ± 1178.8	2549.5 ± 717.1	2342.2 ± 726.0	1275.5 ± 567.6
Parasubiculum	3752.4 ± 1026.5	3349.3 ± 916.4	2704.0 ± 497.6	1397.5 ± 580.2
Entorhinal cortex	3303.6 ± 564.7	3044.3 ± 552.3	3436.0 ± 536.8	1265.0 ± 622.2
**TrkB IR area (μm** ^**2**^ **× 10** ^**2**^ **)**				
Granular layer	1970.3 ± 651.6	3883.8 ± 1442.6	4307.5 ± 1039.8	2591.0 ± 539.0
Hilus	1490.4 ± 551.2	2214.4 ± 712.8	2693.6 ± 838.0	1937.9 ± 510.2
CA4	1369.0 ± 602.3	2590.7 ± 1105.6	2861.6 ± 458.0	2911.2 ± 839.6
CA3	1857.9 ± 436.3	2124.1 ± 703.8	3050.8 ± 646.0	3892.2 ± 958.6
CA2	2311.3 ± 700.8	3276.0 ± 1078.4	3182.0 ± 593.3	3732.2 ± 956.4
CA1	1513.3 ± 442.5	1729.8 ± 540.6	1931.2 ± 596.6	2888.2 ± 585.8
Prosubiculum	1454.7 ± 445.4	1427.5 ± 434.1	2224.4 ± 679.7	2694.9 ± 956.7
Subiculum	1649.9 ± 415.1	2776.0 ± 806.4	2843.5 ± 977.7	2531.9 ± 622.9
Parasubiculum	1689.4 ± 425.3	2833.3 ± 499.8	2778.9 ± 412.3	3000.3 ± 1086.8
Entorhinal cortex	1893.1 ± 302.0	2808.3 ± 509.2	2870.6 ± 588.5	2930.0 ± 857.8
**TrkA IR area (μm** ^**2**^ **× 10** ^**2**^ **)**				
Granular layer	1757.7 ± 646.3	2155.8 ± 721.3	1444.2 ± 289.9	2181.6 ± 889.4
Hilus	1213.1 ± 549.7	1252.2 ± 479.1	1183.7 ± 378.9	1960.9 ± 716.8
CA4	1441.1 ± 553.3	1441.1 ± 470.7	1322.1 ± 291.9	1802.1 ± 570.2
CA3	1473.9 ± 333.1	1643.2 ± 487.0	1193.3 ± 267.3	2614.6 ± 918.1
CA2	1820.7 ± 587.9	2097.6 ± 607.7	1675.1 ± 421.3	2463.7 ± 768.1
CA1	1157.7 ± 350.1	1042.9 ± 291.3	1179.6 ± 462.2	2169.7 ± 637.2
Prosubiculum	1202.9 ± 345.8	1017.0 ± 380.5	1279.3 ± 295.0	1888.3 ± 566.8
Subiculum	1515.8 ± 360.7	1662.1 ± 456.6	1456.9 ± 375.2	1780.5 ± 555.8
Parasubiculum	1733.4 ± 391.2	1524.4 ± 252.6	1572.3 ± 391.2	1834.7 ± 600.3
Entorhinal cortex	1779.4 ± 641.3	1135.7 ± 429.9	1464.5 ± 295.9	1693.8 ± 383.6
**TrkC IR area (μm** ^**2**^ **× 10** ^**2**^ **)**				
Granular layer	1295.3 ± 416.3	1194.4 ± 402.4	1106.0 ± 300.0	1103.5 ± 477.8
Hilus	1120.3 ± 313.4	1114.3 ± 303.8	1076.2 ± 411.7	1427.1 ± 395.4
CA4	1188.5 ± 297.0	1270.0 ± 483.5	1387.8 ± 468.6	1316.9 ± 184.6
CA3	1512.1 ± 425.0	1333.3 ± 787.3	1742.3 ± 608.3	2323.6 ± 772.3
CA2	2076.8 ± 485.9	1630.9 ± 473.3	1808.3 ± 859.2	2012.1 ± 789.9
CA1	1133.3 ± 378.9	1284.6 ± 432.1	1069.6 ± 415.5	1092.8 ± 313.6
Prosubiculum	1200.4 ± 193.7	1069.7 ± 336.3	1083.7 ± 404.3	1117.1 ± 634.2
Subiculum	1261.1 ± 361.2	1158.1 ± 308.1	1172.9 ± 468.8	942.9 ± 154.2
Parasubiculum	1375.7 ± 306.1	1282.1 ± 385.6	1155.6 ± 453.2	1204.2 ± 538.7
Entorhinal cortex	1610.7 ± 438.8	1523.6 ± 522.5	1535.5 ± 598.0	958.1 ± 399.1

Values indicated as mean ± std. deviation or as number of patients (n), when indicated. n.a.: not applicable.

### Clinical features of MTLE patients

All patients were referred for pre-surgical assessment due to drug-resistant seizures as defined in the literature [40]. Patients were evaluated at the Ribeirao Preto Epilepsy Surgery Program using standardized protocols approved by the institution’s Ethics Committee and a written consent form was obtained from each patient. Pre-surgical investigation at the Epilepsy Monitoring Unit included detailed clinical history, neurological examination, interictal and ictal scalp/sphenoidal electroencephalography (EEG), neuropsychology evaluation, and intracarotid amobarbital memory and language procedure whenever deemed clinically necessary.

Definition of MTLE followed Engel’s criteria [41]: (I) Seizure semiology consistent with MTLE, usually with epigastric/autonomic/psychic auras, followed by complex partial seizures; (II) Pre-surgical investigation confirming seizure onset zone in the temporal lobe; (III) Anterior and mesial temporal interictal spikes on EEG; (IV) No lesions other than uni- or bilateral hippocampal atrophy on high resolution magnetic resonance imaging scans (reduced hippocampal dimensions and increased T2 signal); (V) Clinical histopathological examination compatible with HS; (VI) No evidence of dual pathology identifiable by any of the assessment methods described (clinical, electrophysiology, neuroimaging and histopathology). Exclusion criteria were: (I) focal neurological abnormalities on physical examination; (II) generalized or extra-temporal EEG spikes; (III) marked cognitive impairment indicating dysfunction beyond the temporal regions.

Information regarding antecedent of an initial precipitant injury, febrile seizures, seizure types, drug regimen and estimated monthly frequency (within the two years prior to surgery) were retrospectively collected from medical records for each patient. Psychiatric evaluations were conducted in all MTLE patients. Each diagnosis of major depression was independently established during the presurgical evaluation by two psychiatrists with experience in psychiatric disorders associated with epilepsy, using the guidelines of the Diagnostic and Statistical Manual of Mental Disorders, 4th edition. Once a consensus on the classification of psychotic syndromes associated with epilepsy was lacking at the time of patient evaluation and tissue collection, and neither DSM-IV nor ICD-10 had addressed this issue specifically [see review in [34]], the diagnosis of psychosis associated with MTLE was established according to Sachdev [42], meaning that patients with interictal psychosis did not experience the following: psychotic disorder temporally associated with seizures, changes in antiepileptic medications, epileptic status, delirium, and psychosis for paradoxical normalization. This group was defined by a prolonged psychotic state that was not related to the epileptic seizures. Typically, the psychotic states closely resemble schizophrenia, with paranoid ideas which might become systematized, ideas of influence, and auditory hallucinations often of a menacing quality. The points of difference are: common religious coloring of the paranoid ideas, tendency of the affect to remain warm and appropriate, and no typical deterioration to the hebephrenic state [43]. Patients had no history of previous psychiatric disorders (prior to seizure onset) or of substance dependence at any time. Global IQ was calculated after neuropsychological tests (complete WAIS-III or WAIS-R protocol).

### Tissue collection and immunohistochemical processing

Hippocampal body specimens were segmented into 1 cm blocks transversely oriented to the hippocampal long axis and were placed in buffered paraformaldehyde (Sigma, St Louis, MO, USA). After 48–96 hours specimens were processed and paraffin embedded for immunohistochemistry.

Immunohistochemistry was performed with antibodies that identified immunoreactivity for Neu-N, a nuclear protein found in the nuclei of mature neurons (1:1000 dilution; Chemicon-Millipore, Billerica, MA, USA), and for the NT receptors: p75^NTR^, TrkB, TrkA and TrkC (all diluted 1:30; Santa Cruz Biotechnology, Santa Cruz, CA, USA). According to the manufacturers and as already verified in selected references [18,44–48], these antibodies recognize a single band in western blot and show no cross-reactivity with other NT receptors. Briefly, paraffin embedded MTLE and control hippocampi were processed together for each antibody as described in Kandratavicius et al. [7], with overnight incubation at room temperature and developed simultaneously for 10 min in 0.05% 3,3’-diaminobenzidine tetrahydrochloride (Pierce, Rockford, USA) and 0.01% hydrogen peroxide (Merck, Darmstadt, Germany). After sufficient colorization, reaction was halted by washing in several rinses of distilled water, dehydrated through graded ethanol to xylene (Merck, Darmstadt, Germany), and cover slipped with Krystalon (EM Science, Gibbstown, NJ, USA). Adjacent sections were hematoxilin-eosin stained and examined for tissue integrity. Control sections without the primary antisera did not reveal staining (data not shown).

### Cell count

Neuronal counting for MTLE and control hippocampi was performed based on Lorente de No’s classification [49], including fascia dentata granular and subgranular cells, polymorphic hilar neurons (limited to a region between stratum granulosum and CA4 pyramidal cells, being at least 50 μm from the stratum granulosum and 100 μm from CA4), as well as pyramidal cells in CA4 (the portion of Ammon’s horn that permeates the inner part of the dentate gyrus), CA3, CA2, CA1, prosubiculum, subiculum, parasubiculum, and entorhinal cortex layer III. Cell densities (neurons per cubic millimeter) were estimated in 8 μm Neu-N stained slices at 400x magnification with a morphometric grid methodology using Abercrombie’s correction [50] as previously described and well established in the literature for surgical hippocampal fragments [3,5–7,51–55].

### Semi-quantitative analysis of immunohistochemistry

Adjacent slides to those examined for neuronal density were analyzed for NT receptors expression. In brief, all digitized images were analyzed with Image J software, following the same criteria: (I) the software identifies the gray value distribution of a subfield’s digital image (total area image for each subfield = 313.7 μm × 235.3 μm); (II) the immunoreactive (IR) area is selected (i.e. positive stained pixels), limited to a threshold range; and (III) the threshold range is pre-settled based on control group sections, to exclude the low intensity gray value of background staining from the analysis. A similar approach was used by our group elsewhere [6]. Results for granular layer included granular cell layer per se and proximal molecular layer. Analyses were conducted by one investigator (LK), blind to hippocampal pathology and group classification.

### Data analysis

Data were analyzed using the statistical program PAWS (version 18.0) and SigmaPlot (version 11.0). Groups were compared using analysis of variance (ANOVA one way, with Bonferroni post-hoc test) or unpaired *t* test for variables with normal distribution, and Kruskal-Wallis One Way Analysis of Variance on Ranks (with Dunn post hoc test) or Mann–Whitney Rank Sum Test for variables without normal distribution. Fisher Exact test was applied for comparison of relative frequencies of clinical variables between groups. Other statistical tests included two-way ANOVA with Holm-Sidak post-hoc test, Pearson correlation analyses and analysis of covariance (ANCOVA). Statistical significance was set at p < 0.05 and values presented as mean ± SD. Depicted p values in all ANOVA analysis refer to p values after post-hoc tests.

## Results

### Clinical profiles

The four patients groups did not show significant differences in gender, age or collected side (Table 1). Clinical variables such as presence of an initial precipitant injury and febrile seizures, age of first seizure and seizure onset, seizure frequency and epilepsy duration, HS side, handedness, IQ, years at school and performance in verbal memory tests were homogeneously distributed among MTLE groups. MTLE + D patients exhibited a trend (0.05 ≤ *p* ≤ 0.07) to increased proportion of patients with secondarily generalized seizures (SGS) when compared to patients without psychiatric comorbidities, and MTLE + P patients exhibited a trend to worse performance in non-verbal memory tasks.

Two patients in MTLE group, one patient in MTLE + D, and four patients in MTLE + P group presented with auras and seizures post-surgery, although frequency was decreased if compared with pre-surgery period. The remaining of patients without complete remission had only auras, and the proportion of patients with this outcome was higher in the group with history of major depression when compared to MTLE without psychiatric comorbidities (Fisher’s Exact Test, p = 0.046).

All epileptic patients were on antiepileptic drugs (carbamazepine, oxcarbazepine, phenobarbital, and/or phenytoin). In addition, patients were also taking benzodiazepines (MTLE group: 8 of 14; MTLE + D: 10 of 13; MTLE + P group 10 of 13), fluoxetine (MTLE + D: 4 of 13) and haloperidol (MTLE + P group: 10 of 13). No differences in neuronal density, or NT receptors expression were seen between patients taking or not taking benzodiazepines. No differences in neuropsychological tests between patients taking or not taking benzodiazepines, fluoxetine or haloperidol were seen. Possible influence of fluoxetine and haloperidol in NT receptors expression will be explored on the next sections.

### Neuropathological characterization: neuronal density

**Figure 1:**
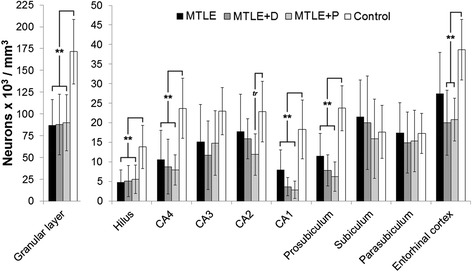
**Neuronal density in human hippocampal formation subfields.** Neuronal density values from MTLE (black bars), MTLE + D (gray bars), MTLE + P (light gray bars) and from non-epileptic controls (white bars) are indicated as mean ± std. deviation. Double asterisk indicate significant statistical difference (*p* < 0.001) between epileptics and control group. Neuronal loss was observed in the granular layer, hilus, CA4, CA1, prosubiculum and entorhinal cortex. A statistical trend (*tr*: 0.05 ≤ *p* ≤ 0.07) to decreased neuronal density in MTLE + P CA2 when compared to control can also be seen.

Evaluation of epileptogenic and control hippocampal formation showed reduced neuron density in the granular layer, hilus, CA4, CA1 and prosubiculum of all MTLE groups when compared to control (Figure 1, asterisks). In addition, we found significant neuron density reduction in the entorhinal cortex of MTLE patients with major depression and interictal psychosis when compared to controls and a trend to decreased neuronal density in CA2 of MTLE + P specimens. More details on neuronal density and its relation with clinical features can be found in our previous study on NTs expression [9], since we used here the same specimens’ series. Results of neuronal densities were showed here again because given the differences among the groups, an important question was whether the statistical differences in NT receptors expression for the four groups could be accounted for by changes in neuronal densities. We therefore performed an analysis of covariance (ANCOVA) comparing groups and neuronal densities with NT receptors IR area and the results are shown in Table 3. As depicted, difference between groups in NT receptors expression remained significant in all subfields for TrkB, five subfields for p75^NTR^ and two subfields for TrkA after cell count correction (Table 3, boldfaced values). These results indicate that the groups showed significant differences in NT receptors expression levels that were not influenced by changes in neuronal densities.

**Table 3:** **Analysis of Covariance (ANCOVA) comparing patients’ categories and neuronal density (counts), with NT receptors IR area**

**Subfield**	***p75*** ^***NTR***^ ***(counts as covariable)***	***TrKA (counts as covariable)***	***TrkB (counts as covariable)***	***TrkC (counts as covariable)***
Granular layer				
	2.748/0.060	1.369/0.275	**12.234/0.000**	0.778/0.516
Hilus				
	2.618/0.075	1.087/0.375	**4.305/0.013**	0.320/0.811
CA4				
	**5.551/0.006**	0.119/0.948	**4.864/0.010**	0.058/0.981
CA3				
	3.333/0.056	2.426/0.116	**9.452/0.001**	1.616/0.238
CA2				
	2.531/0.090	1.248/0.333	**3.693/0.033**	0.757/0.534
CA1				
	**3.281/0.037**	**7.080/0.002**	**6.741/0.002**	0.077/0.972
Prosubiculum				
	1.454/0.259	**3.798/0.026**	**3.946/0.022**	0.826/0.497
Subiculum				
	**6.252/0.002**	2.162/0.117	**5.724/0.003**	1.346/0.282
Parasubiculum				
	**10.676/0.000**	2.877/0.058	**7.286/0.001**	0.368/0.777
Entorhinal cortex				
	**8.094/0.003**	1.377/0.297	**6.030/0.010**	2.399/0.119

Data are presented as F values/p values, and significant results are indicated in boldfaced type.

### NT receptors expression

**Figure 2:**
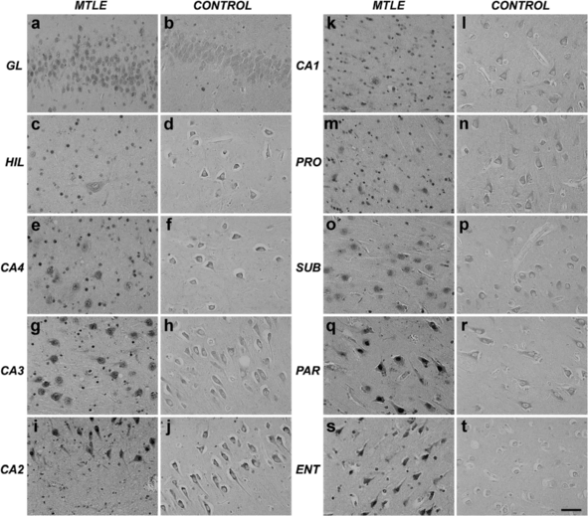
**p75** ^**NTR**^ **expression in the human hippocampal formation.** Increased expression can be seen in MTLE neurons and glial-like cells, while in control only neurons are stained. GL: granular layer; HIL: hilus; PRO: prosubiculum; SUB: subiculum; PAR: parasubiculum; ENT: entorhinal cortex. *Scale bar* **(a-t)**: 50 μm.

**Figure 3:**
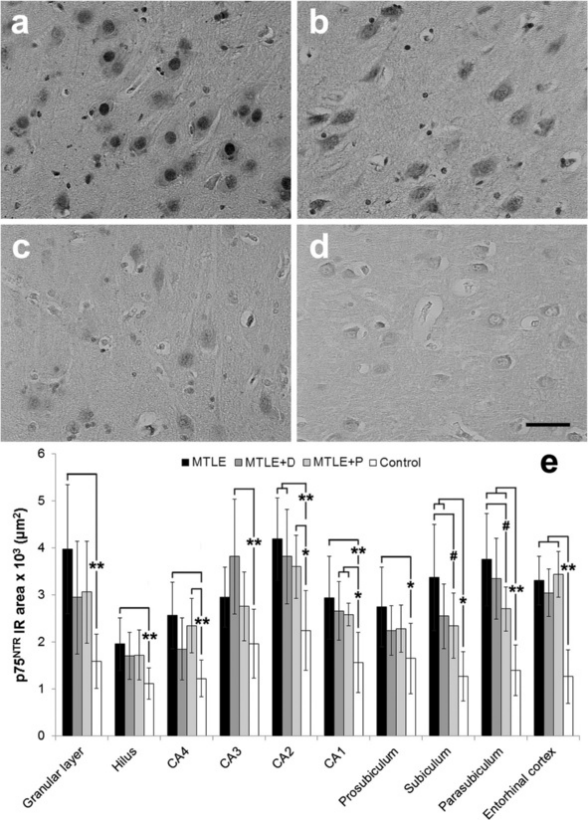
**p75** ^**NTR**^ **expression in MTLE specimens with and without psychiatric comorbidities and in non-epileptic controls.** MTLE subiculum **(a)** exhibited increased p75^NTR^ expression when compared to MTLE + D **(b)**, MTLE + P **(c)** and control group **(d)**. We found p75^NTR^ immunoreactive area values **(e)** higher epileptic groups than in controls (double asterisk, *p* < 0.01; single asterisk, *p* < 0.05), and differences among MTLE groups (single hash sign, *p* < 0.05). Values from MTLE (black bars), MTLE + D (gray bars), MTLE + P (light gray bars) and from non-epileptic controls (white bars) are indicated as mean ± std. deviation. *Scale bar* **(a-d)**: 50 μm.

Strong p75^NTR^ immunoreactivity was detected in all hippocampal formation subfields (Figure 2, a-t). In MTLE specimens, glial staining was evident, while in controls only neurons were immunoreactive (IR). In subiculum, increased staining was seen in MTLE specimens, and less in MTLE with psychosis and controls (Figure 3, a-d). p75^NTR^ IR area was lower in controls (Figure 3e, asterisks) and independent of neuronal density in CA4, CA1, subiculum, parasubiculum and entorhinal cortex (Table 3). Among MTLE groups, patients with interictal psychosis showed decreased p75^NTR^ expression in subiculum and parasubiculum (Figure 3e, hash signs).

Patients with SGS showed decreased p75^NTR^ expression in all hippocampal formation subfields when compared to those with complex partial seizures (CPS), and the difference was statistically significant in the granular layer (SGS: 3030.8 ± 1232.7 × 10^2^ μm^2^ versus CPS: 4200.1 ± 1271.1 × 10^2^ μm^2^, *t* (38) = 2.51, *p* = 0.02), CA4 (SGS: 2073.3 ± 693.0 × 10^2^ μm^2^ versus CPS: 2723.7 ± 664.9 × 10^2^ μm^2^, *t* (35) = 2.25, *p* = 0.03), and CA2 (SGS: 3611.0 ± 833.5 × 10^2^ μm^2^ versus CPS: 4631.6 ± 725.9 × 10^2^ μm^2^, *t* (32) = 2.82, *p* = 0.01). No interaction between psychiatric status and seizure type was seen (*p* = 0.875). No effects of fluoxetine or haloperidol were seen on p75^NTR^ expression.

**Figure 4:**
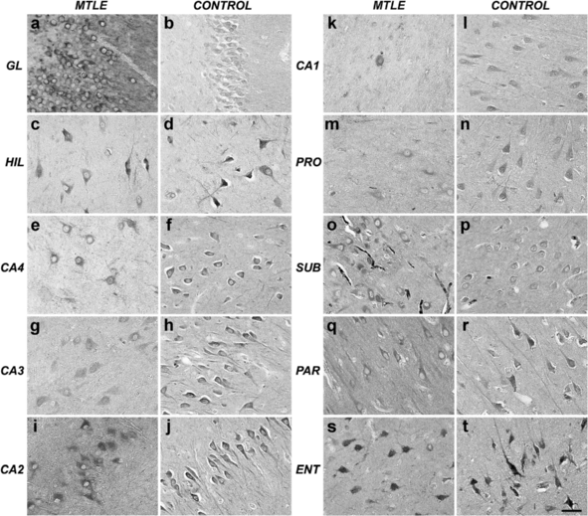
**TrkB expression in the human hippocampal formation.** Predominant neuronal expression can be seen in all subfields. Apical dendrites were also evident in MTLE and controls. GL: granular layer; HIL: hilus; PRO: prosubiculum; SUB: subiculum; PAR: parasubiculum; ENT: entorhinal cortex. *Scale bar* **(a-t)**: 50 μm.

**Figure 5:**
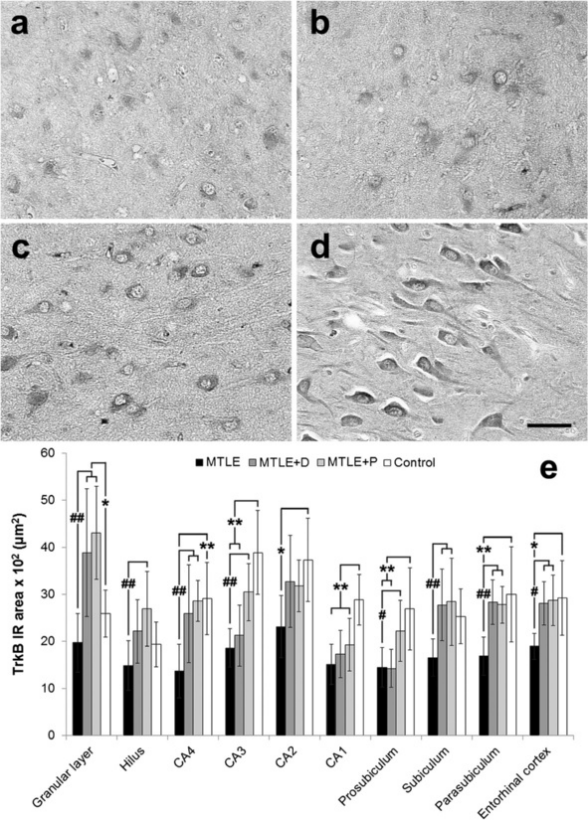
**TrkB expression in MTLE specimens with and without psychiatric comorbidities and in non-epileptic controls.** MTLE **(a)** and MTLE + D **(c)** exhibited decreased TrkB expression in CA3 when compared to MTLE + P **(b)** and control group **(d)**. We found TrkB immunoreactive area values **(e)** higher in the dentate gyrus of epileptic groups with psychiatric comorbidities, and decreased in other subfields of MTLE specimens when compared to controls (double asterisk, *p* < 0.01; single asterisk, *p* < 0.05). Differences among epileptic groups were seen in the granular layer, hilus, CA4, CA3, prosubiculum, subiculum, parasubiculum and entorhinal cortex, where MTLE specimens showed decreased TrkB expression when compared to MTLE with psychiatric comorbidities (single hash sign, *p* < 0.05; double hash sign, *p* < 0.01). Values from MTLE (black bars), MTLE + D (gray bars), MTLE + P (light gray bars) and from non-epileptic controls (white bars) are indicated as mean ± std. deviation. *Scale bar* **(a-d)**: 50 μm.

TrkB expression in neurons and dendritic processes was detected in all subfields of the hippocampal formation (Figure 4, a-s). In CA3, increased TrkB IR was seen in MTLE + P and controls when compared to MTLE and MTLE + D (Figure 5, a-d). Among MTLE groups, MTLE without psychiatric comorbidities showed consistently lower TrkB expression (Figure 5e, hash signs), with exception of CA2 and CA1, where differences did not reach statistical significance. In the granular layer, CA4, subiculum, parasubiculum and entorhinal cortex both MTLE + D and MTLE + P presented with increased TrkB expression when compared to MTLE without psychiatric comorbidities. Controls showed higher TrkB expression than MTLE in the entire Ammon’s horn, prosubiculum, parasubiculum and entorhinal cortex (Figure 5e, asterisks).

Interestingly, TrkB expression in patients with SGS was higher than in patients with CPS in all hippocampal formation subfields, being statistically significant in CA4 (SGS: 2487.0 ± 961.9 × 10^2^ μm^2^ versus CPS: 1640.5 ± 835.7 × 10^2^ μm^2^, *t* (35) = −2.29, *p* = 0.03). No interaction between psychiatric status and seizure type was seen (*p* = 0.711) suggesting that increased TrkB expression levels seen in specimens from patients with a psychiatric diagnosis as well as on those with SGS are independent findings. Moreover, complete seizure remission post-surgery was achieved by the ones with lower hippocampal TrkB expression. Difference between groups was significant in the granular layer (Remission: 2768.2 ± 1086.5 × 10^2^ μm^2^ versus No-remission: 4418.1 ± 1864.7 × 10^2^ μm^2^, *t* (38) = 2.89, *p* = 0.008) and prosubiculum (Remission: 1533.1 ± 456.0 × 10^2^ μm^2^ versus No-remission: 2179.9 ± 688.4 × 10^2^ μm^2^, *t* (36) = 2.61, *p* = 0.02). Psychiatric status and seizure outcome exhibited a significant interaction (*p* = 0.016), probably due to an increased proportion of patients without complete seizure remission and with a psychiatric diagnosis. In fact, differences in TrkB expression between remission versus no-remission patients were evident in MTLE with psychiatric comorbidities (*p* < 0.007) but not on those without psychiatric comorbidities (*p* = 0.717). Also, a trend to a direct correlation between seizure frequency and TrkB expression in the granular layer was noted (Pearson’s R = +0.35, p = 0.05). No effects of fluoxetine or haloperidol were seen on TrkB expression.

**Figure 6:**
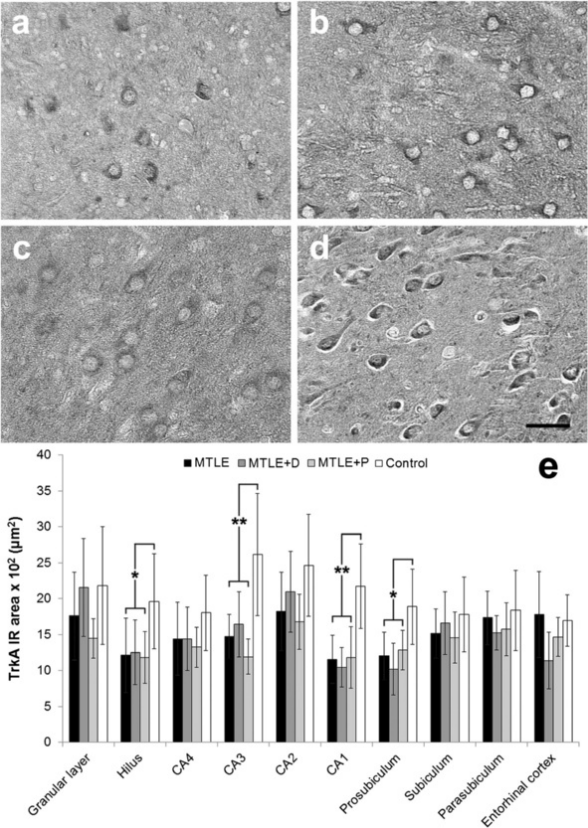
**TrkA expression in MTLE specimens with and without psychiatric comorbidities and in non-epileptic controls.** MTLE **(a)**, MTLE + D **(b)** and MTLE + P **(c)** exhibited decreased TrkA expression in CA3 when compared to control group **(d)**. We found TrkA immunoreactive area values **(e)** lower in the hilus, CA3, CA1 and prosubiculum of epileptic groups when compared to controls (double asterisk, *p* < 0.01; single asterisk, *p* < 0.05). Values from MTLE (black bars), MTLE + D (gray bars), MTLE + P (light gray bars) and from non-epileptic controls (white bars) are indicated as mean ± std. deviation. *Scale bar* **(a-d)**: 50 μm.

TrkA expression was predominantly neuronal (Figure 6, a-d) and decreased in epileptic groups when compared to controls in the hilus, CA3, CA1 and prosubiculum (Figure 6e, asterisks). When corrected for neuronal densities differences between MTLE groups and control, CA1 and prosubiculum remained significant (Table 3). No differences among MTLE groups or in seizure type and outcome were seen regarding TrkA levels. In MTLE + D group, patients taking fluoxetine showed increased TrkA expression in CA4 (1804.1 ± 366.8 × 10^2^ μm^2^ versus 1088.8 ± 309.6 × 10^2^ μm^2^, *t* (10) = 2.81, *p* = 0.04) and CA2 (2601.7 ± 583.0 × 10^2^ μm^2^ versus 1719.5 ± 259.2 × 10^2^ μm^2^, *t* (9) = 2.75, *p* = 0.04). In MTLE + P group, patients taking haloperidol showed decreased TrkA expression in CA4 (1155.7 ± 164.9 × 10^2^ μm^2^ versus 1654.9 ± 153.7 × 10^2^ μm^2^, *t* (11) = −4.36, *p* = 0.003).

**Figure 7:**
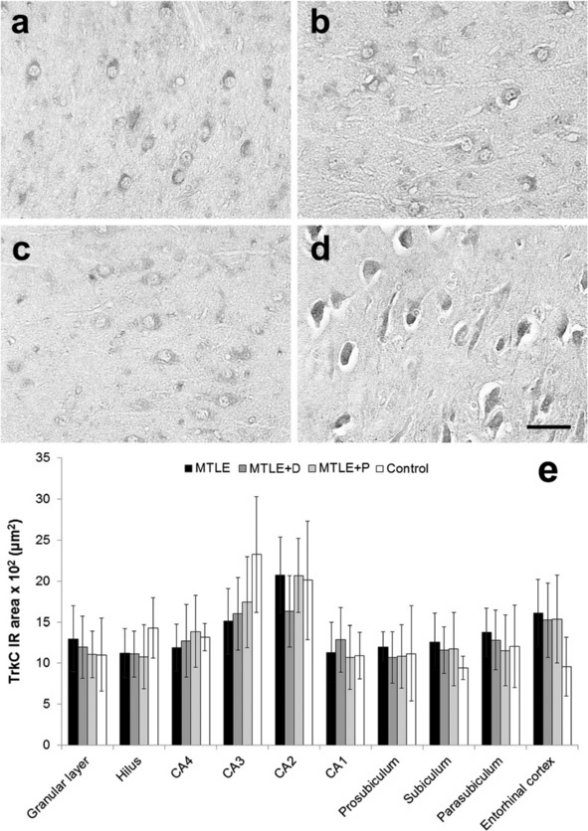
**TrkC expression in MTLE specimens with and without psychiatric comorbidities and in non-epileptic controls.** MTLE **(a)** and MTLE + D **(b)**, MTLE + P **(c)** and control group **(d)** exhibited predominatly neuronal TrkC expression in CA3. No statistical differences in TrkC immunoreactive area were found among groups **(e)**. Values from MTLE (black bars), MTLE + D (gray bars), MTLE + P (light gray bars) and from non-epileptic controls (white bars) are indicated as mean ± std. deviation. *Scale bar* **(a-d)**: 50 μm.

TrkC expression was also predominantly neuronal (Figure 7, a-d) but no differences among groups were found (Figure 7e). In MTLE + D group, patients taking fluoxetine showed increased TrkC expression in CA4 (1741.8 ± 88.7 × 10^2^ μm^2^ versus 1034.0 ± 405.3 × 10^2^ μm^2^, *t* (11) = 3.51, *p* = 0.03). In order to examine whether there was an imbalance in the expression of TrkA, B, and C in relation to p75^NTR^ expression, we determined the expression ratios of p75^NTR^ versus TrkA, TrkB, and TrkC. As shown in Table 4, we observed significant increase in expression ratios of p75^NTR^ versus TrkA in all subfields except CA3 and prosubiculum. p75^NTR^/TrkB ratios were elevated in all hippocampal formation subfields of epileptogenic hippocampus when compared to controls. Besides, differences among MTLE groups were also detected in all subfields except CA3, CA2 and CA1, with decreased p75^NTR^/TrkB ratio in MTLE with psychiatric comorbidities. Imbalance in the expression of TrkB in relation to p75^NTR^ expression may also play a role in seizure type since patients with SGS showed decreased p75^NTR^/TrkB ratio when compared to those with CPS. Difference between groups was significant in the granular layer (SGS: 1.13 ± 0.75 versus CPS: 1.86 ± 1.07, *t* (38) = 2.25, *p* = 0.03), CA4 (SGS: 0.98 ± 0.51 versus CPS: 2.13 ± 1.13, *t* (35) = 2.59, *p* = 0.02), CA1 (SGS: 1.55 ± 0.50 versus CPS: 2.31 ± 0.78, *t* (36) = 3.11, *p* = 0.005), and prosubiculum (SGS: 1.34 ± 0.48 versus CPS: 1.90 ± 0.59, *t* (36) = 2.54, *p* = 0.02). No interaction between psychiatric status and seizure type was seen (*p* = 0.867), suggesting that decreased p75/TrkB ratio levels detected in specimens from patients with a psychiatric diagnosis as well as on those with SGS are independent findings. Expression ratios of p75^NTR^ to TrkC were increased in MTLE patients when compared to controls in the dentate gyrus, CA3, CA2, parasubiculum and entorhinal cortex.

**Table 4:** **Ratios of p75** ^**NTR**^ **/Trks in hippocampal subfields of MTLE groups and normal controls**

	**MTLE**	**MTLE + D**	**MTLE + P**	**Controls**	**p value**
**TrkA**					
Granular layer	2.4 ± 1.1	1.4 ± 0.4	2.1 ± 1.4	1.0 ± 0.5	*0.04* (MTLE × CTRL)
Hilus	2.3 ± 2.0	1.4 ± 0.5	1.5 ± 0.5	0.8 ± 0.4	*0.04* (MTLE × CTRL)
CA4	1.9 ± 0.8	1.4 ± 0.4	1.9 ± 0.6	0.9 ± 0.3	*0.04* (MTLE and MTLE + P × CTRL)
CA3	1.9 ± 1.0	2.3 ± 0.9	2.6 ± 1.0	1.1 ± 0.6	n.s.
CA2	2.6 ± 1.2	1.7 ± 0.5	2.1 ± 0.6	1.1 ± 0.6	*0.04* (MTLE x CTRL)
CA1	2.6 ± 1.4	2.6 ± 0.9	2.3 ± 1.1	0.9 ± 0.5	*0.04* (MTLE and MTLE + D × CTRL)
Prosubiculum	2.1 ± 1.2	2.1 ± 1.0	1.9 ± 0.3	1.0 ± 0.4	n.s.
Subiculum	2.3 ± 1.0	1.5 ± 0.5	1.8 ± 0.6	0.9 ± 0.5	*0.005* (MTLE x CTRL)
Parasubiculum	2.2 ± 0.6	2.3 ± 0.6	1.8 ± 0.4	1.1 ± 0.5	*0.004* (MTLE and MTLE + D × CTRL)
Entorhinal cortex	2.3 ± 0.8	3.5 ± 2.1	2.4 ± 1.0	0.9 ± 0.4	*0.02* (MTLE + D × CTRL)
**TrkB**					
Granular layer	2.2 ± 0.9	0.9 ± 0.5	0.8 ± 0.5	0.6 ± 0.2	*<0.0001* (MTLE × all groups)
Hilus	1.6 ± 1.1	0.9 ± 0.4	0.7 ± 0.2	0.6 ± 0.1	*0.01* (MTLE × MTLE + P); *0.02* (MTLE × CTRL)
CA4	2.1 ± 1.0	0.9 ± 0.4	0.8 ± 0.2	0.5 ± 0.1	*<0.003* (MTLE × all groups)
CA3	1.7 ± 0.9	2.0 ± 1.0	1.0 ± 0.4	0.6 ± 0.2	*0.04* (MTLE and MTLE + D × CTRL)
CA2	2.1 ± 1.3	1.3 ± 0.4	1.1 ± 0.2	0.6 ± 0.1	*0.01* (MTLE × CTRL)
CA1	2.1 ± 0.8	1.5 ± 0.4	1.5 ± 0.5	0.6 ± 0.2	*<0.0001* (MTLE × CTRL)
Prosubiculum	2.1 ± 0.5	1.4 ± 0.4	1.2 ± 0.3	0.7 ± 0.1	*<0.01* (MTLE × all groups); *0.03* (MTLE + D × CTRL)
Subiculum	2.1 ± 0.8	1.0 ± 0.4	0.9 ± 0.3	0.6 ± 0.3	*<0.0001* (MTLE × all groups)
Parasubiculum	2.3 ± 0.7	1.2 ± 0.4	1.0 ± 0.2	0.6 ± 0.2	*<0.0001* (MTLE × all groups)
Entorhinal cortex	1.9 ± 0.4	1.1 ± 0.4	1.3 ± 0.4	0.5 ± 0.1	*0.01* (MTLE × MTLE + D); <*0.01* (MTLE groups × CTRL)
**TrkC**					
Granular layer	3.4 ± 1.7	3.0 ± 1.1	2.9 ± 1.2	1.6 ± 0.7	<*0.01* (MTLE groups × CTRL)
Hilus	2.0 ± 1.0	1.5 ± 0.5	1.6 ± 0.3	0.8 ± 0.2	*0.009* (MTLE × CTRL)
CA4	2.3 ± 1.2	1.6 ± 0.6	1.7 ± 0.6	1.0 ± 0.4	n.s.
CA3	2.0 ± 0.7	2.4 ± 0.4	1.7 ± 0.4	1.1 ± 0.5	*<0.01* (MTLE and MTLE + D × CTRL)
CA2	2.2 ± 0.8	2.4 ± 0.4	1.7 ± 0.4	1.1 ± 0.5	*0.02* (MTLE and MTLE + D × CTRL)
CA1	2.9 ± 1.3	2.7 ± 1.8	2.4 ± 0.7	1.3 ± 0.7	n.s.
Prosubiculum	2.2 ± 1.1	1.7 ± 0.4	2.2 ± 1.0	1.7 ± 1.2	n.s.
Subiculum	2.7 ± 1.1	2.1 ± 0.5	2.2 ± 1.3	1.1 ± 0.3	n.s.
Parasubiculum	2.7 ± 0.5	2.7 ± 0.5	2.4 ± 0.8	1.3 ± 0.5	*<0.01* (MTLE groups × CTRL)
Entorhinal cortex	2.2 ± 0.5	2.4 ± 0.9	2.9 ± 1.0	1.1 ± 0.5	*0.03* (MTLE + P × CTRL)

Values indicated as mean ± std. deviation. n.s.: non-significant.

## Discussion

In the present study, we found in the epileptogenic hippocampal formation increased expression of p75^NTR^, decreased TrkA expression, and complex alterations involving TrkB expression. Increased TrkB expression in patients without complete seizure remission and in those with SGS, and a trend to a direct correlation between TrkB and seizure frequency was seen. In addition, decreased p75^NTR^ expression associated with interictal psychosis, and increased TrkB in those with psychosis or major depression was also reported, although their p75^NTR^/TrkB ratios were lower than in MTLE without psychiatric comorbidities.

There are scarce reports on hippocampal NT receptors expression in schizophrenia and mood disorders, showing unaltered, decreased and increased levels [31,56,57]. Since our series refers to psychiatric states comorbid with epilepsy, our discussion will focus mainly in NT receptors expression considering whenever possible evidences found in epilepsy. In our earlier study [9], we found increased nerve growth factor (NGF) and brain derived neurotrophic factor (BDNF) expression in MTLE, and decreased NGF and BDNF in specific hippocampal subfields of MTLE + P, but increased NGF in the granular layer of MTLE + D. Those previous and the present results can be directly correlated since they are from the same patient’s series. In MTLE and MTLE + D, a decrease in TrkA might represent a compensatory effect to increased NGF. Since in MTLE + P both NGF and TrkA are in low levels, we could expect a diminished trophic tone, for instance, in mossy fiber sprouting, as we indeed noticed in previous studies [7,9]. BDNF was also decreased in MTLE + P granular layer [9], but its high affinity ligand TrkB was increased. Here we must emphasize the relevance of our expression ratios’ findings between p75^NTR^ and Trks. The low affinity NT receptor p75^NTR^ may have a pro-apoptotic role when acting without Trks and/or as a ligand for pro-NTs; and p75^NTR^ may have an anti-apoptotic role, strengthening mature NT biding and signal transduction when forming a complex with Trks [58]. Coexpression of p75^NTR^ and Trks has been previously demonstrated with immunofluorescence in hippocampal sclerosis [20], and a way to quantitatively express this relation is by examining p75^NTR^ and Trks expression ratios, as explored by Dwivedi et al. [32]. Increased p75^NTR^/Trks ratios in most hippocampal subfields of MTLE specimens suggests mechanisms toward cell survival and enhancement of the efficacy of synaptic neurotransmission, but decreased p75^NTR^/TrkB ratios in MTLE with psychiatric comorbidities could be associated with structural abnormalities and reduced neuronal plasticity in these hippocampi. In fact, significant neuronal loss in the entorhinal cortex and a trend in CA2 were seen only in specimens with major depression and/or psychosis.

In schizophrenia, decreased expression of TrkB in the prefrontal cortex may contribute to reduced BDNF-TrkB signaling and lead to reduced neuronal plasticity [26,29]. In our previous study [9] we hypothesized that given the reciprocal BDNF/glutamate modulation [59], normalization of low BDNF levels found in interictal psychosis [9] would help to reestablish the balance in a possible situation of glutamatergic hypofunction. As reviewed by Schuman [60], TrkB signaling becomes readily desensitized after binding BDNF. Thus, BDNF-TrkB signaling could be potentiated in presence of increased TrkB availability, as it seems to occur in MTLE + P. Together with the already increased BDNF baseline expression found in epilepsy, a comorbid psychotic state would display milder symptoms than in schizophrenia, as it is indeed depicted in interictal psychosis [43]. In major depression we found equally increased TrkB levels as in psychosis, and interestingly, another study showed that NT receptors expression levels were not different between suicide subjects with major depression or with a history of other psychiatric disorders [32]. Although increased TrkB expression in MTLE + D could be an effect of antidepressant drug exposure as extensively documented in the literature [61,62], in our series we found an effect of fluoxetine only on TrkA and TrkC expression. Brain specimens of patients with schizophrenia and major depression usually show decreased TrkB expression, although there are also findings of unaltered levels [31,63]. Our results have indicated that increased TrkB expression in hippocampal specimens of MTLE with psychiatric comorbidities and in those with SGS are independent findings, and we should reemphasize that we are examining psychiatric states secondary to epilepsy. Therefore, if TrkB is intrinsically related to hyperexcitability, epileptogenesis and seizures [19,64], a comorbid psychiatric condition in epilepsy could display a different pattern of expression when compared to the pure forms of the psychopathologies, as we found in our results.

Glial expression of p75^NTR^ was detected in MTLE groups, as already described in human MTLE [20] and in seizure models [65]. While in hippocampal neurons NGF binding to p75^NTR^ results in apoptosis [66], in glial cells the signal may serve as a negative modulator to restrict its proliferation [65]. If there is a differential glial expression and activation in MTLE with and without psychiatric comorbidities as suggested by our NTs expression results, it needs to be further clarified.

Just recently the clinical correlates of SGS in temporal lobe epilepsy were described. Bone et al. [67] reported a positive association between the presence of hippocampal sclerosis and SGS, which is in agreement with the high proportion of patients with SGS in our series. Nevertheless, SGSs are not uniform entities [68], and the pathophysiology of secondary generalization is still poorly understood. We found that patients with SGS showed increased TrkB expression, decreased p75^NTR^/TrkB ratio, and decreased p75^NTR^ expression when compared to those with CPS. Also, there was a trend to a direct correlation between seizure frequency and TrkB expression. The close relationship of TrkB and epileptogenesis has been explored in animal models for more than a decade [17,19,22,23,69,70], and TrkB acts as an indispensable molecule for seizure generation and epilepsy development. Here we described for the first time in human MTLE that even in a chronic scenario TrkB may play a major role on seizure type and epilepsy surgery outcome. Regarding lower p75^NTR^ levels found in SGS it is useful to recollect that we found decreased NGF in those same specimens [9]. In the kindling model, when a synthetic peptide is used to interfere with NGF binding to TrkA and p75^NTR^ receptors, the kindling process is suppressed [21]. In fact, when NGF is not able to properly interact with their receptors, seizure development is halted [71]. In chronic MTLE, lower levels of NGF, TrkA and p75^NTR^ might function as an endogenous mechanism counteracting increased excitability.

A limitation of the current study is the relative heterogeneity of psychiatric groups. We were not able to determine quantitative details of depressive and psychotic symptoms such as precise number and duration of episodes, although all patients in MTLE + D and MTLE + P groups had a lifetime history of their respective psychiatric comorbidity. There are few studies examining neuropathological correlates in the hippocampus of patients with epilepsy and psychiatric comorbidities, and more detailed studies using psychometric scales are still needed. Also, in spite of the fact that we could not perform stereological counts due to limited tissue source from surgery, our neuron density numbers are in agreement with recent hippocampal stereological counts performed in MTLE specimens [72]. In fact, since all MTLE surgical specimens were submitted to identical processing, differences among them are particularly relevant. As with any human study, assumptions must be considered when interpreting our results. Receptor expression does not necessarily equal functional activity, but given our results and evidences in the literature we assumed that such a relationship could exist in some circumstances. Despite our relatively small sample size, other studies exploring neuropathological aspects of different psychiatric comorbidities of epilepsy have been using even smaller series [73,74], and our results are in agreement with the recent NT receptors’ literature.

## Conclusion

Our previous studies [7–9] together with the present results demonstrate that there is an overall structural abnormality and alterations in expression of NTs and their receptors in the epileptogenic hippocampus, that are differentially modulated in presence of psychiatric comorbidities. The clinical significance of these alterations is yet being established. NTs and their receptors are able to regulate central neurotransmission and promote neuroplasticity, representing a promising study area for the development of new treatment strategies, clearly needed in face of different clinical symptoms and differentially affected neuropathological substrates.
